# Genomic and epigenomic predictors of response to guadecitabine in relapsed/refractory acute myelogenous leukemia

**DOI:** 10.1186/s13148-019-0704-3

**Published:** 2019-07-22

**Authors:** Woonbok Chung, Andrew D. Kelly, Patricia Kropf, Henry Fung, Jaroslav Jelinek, Xiang Yao Su, Gail J. Roboz, Hagop M. Kantarjian, Mohammad Azab, Jean-Pierre J. Issa

**Affiliations:** 1 Fels Institute for Cancer Research and Molecular Biology, Temple University School of Medicine, Philadelphia, PA USA; 2 Fox Chase Cancer Center, Temple Health, Philadelphia, PA USA; 3 Astex Pharmaceuticals Inc., Pleasanton, CA USA; 4 Weill Cornell Medicine, Division of Hematology and Oncology, The New York Presbyterian Hospital, New York, NY USA; 5 MD Anderson Cancer Center, Houston, TX USA; 6 Present address: Coriell Institute for Medical Research, 403 Haddon Ave, Camden, NJ 08103 USA

**Keywords:** AML, Guadecitabine, Mutations, DNA methylation inhibitor, Drug resistance, Gene expression

## Abstract

**Background:**

Guadecitabine is a novel DNA methyltransferase (DNMT) inhibitor with improved pharmacokinetics and clinical activity in a subset of patients with relapsed/refractory acute myeloid leukemia (r/r AML), but identification of this subset remains difficult.

**Methods:**

To search for biomarkers of response, we measured genome-wide DNA methylation, mutations of 54 genes, and expression of a panel of 7 genes in pre-treatment samples from 128 patients treated at therapeutic doses in a phase I/II study.

**Results:**

Response rate to guadecitabine was 17% (2 complete remission (CR), 3 CR with incomplete blood count recovery (CRi), or CR with incomplete platelets recovery (CRp)) in the phase I component and 23% (14 CR, 9 CRi/CRp) in phase II. There were no strong mutation or methylation predictors of response. Gene expression clustering defined a subset of patients (~ 20%) that had (i) high DNMT3B and low CDKN2B, CTCF, and CDA expression; (ii) enrichment for KRAS/NRAS mutations; (iii) frequent CpG island hypermethylation; (iv) low long interspersed nuclear element 1 (LINE-1) hypomethylation after treatment; and (v) resistance to guadecitabine in both phase I (response rate 0% vs. 33%, *p* = 0.07) and phase II components of the study (response rate 5% vs. 30%, *p* = 0.02). Multivariate analysis identified peripheral blood (PB) blasts and hemoglobin as predictors of response and cytogenetics, gene expression, RAS mutations, and hemoglobin as predictors of survival.

**Conclusions:**

A subset of patients (~ 20%) with r/r AML is unlikely to benefit from guadecitabine as a single agent. In the remaining 80%, guadecitabine is a viable option with a median survival of 8 months and a 2-year survival rate of 21%.

**Trial registration:**

NCT01261312.

**Electronic supplementary material:**

The online version of this article (10.1186/s13148-019-0704-3) contains supplementary material, which is available to authorized users.

## Introduction

Acute myeloid leukemia (AML) is a highly lethal hematopoietic malignancy characterized by poor long-term survival and relatively few somatic mutations compared to other cancer types [1, 2]. Interestingly, the most commonly mutated genes in AML are enriched for epigenetic regulators such as DNA methyltransferase 3A (DNMT3A), Tet methylcytosine dioxygenase 2 (TET2), and isocitrate dehydrogenase (IDH) 1/2 [3]. In addition, DNA methylation in AML has been shown to harbor disruptions compared to normal blood [4], and various epigenetic signatures have been associated with differential chemotherapy response and prognosis [5]. At present, there are two hypomethylating agents (HMAs)—azacitidine and decitabine—which have been FDA approved for myelodysplastic syndromes (MDS) and are recommended for AML in patients who cannot tolerate intensive chemotherapy [6].

Current HMAs are considered low-intensity therapy in AML and function by blocking the activity of DNMT1, which in turn cannot copy DNA methylation across cell divisions. Although some improvements in remission and overall survival (OS) have been observed in MDS and AML, durable responses are rare, and one potential limitation is the low plasma half-life of these agents [7]. To address this issue, guadecitabine (formerly known as SGI-110) was developed to be resistant to degradation by cytidine deaminase (CDA) with a gradual release of active metabolite decitabine thereby exposing malignant cells to the active drug for longer. Guadecitabine is currently in several phase III clinical trials for AML and MDS after having shown promising phase I/II results [8–10].

Although HMAs such as guadecitabine induce remissions in AML, there are no validated biomarkers that can be used to select patients for therapy. This is especially relevant in relapsed AML, where the response rates are relatively low. In this study, we examined blood and/or bone marrow samples from relapsed/refractory AML (r/r AML) patients enrolled in phase I/II trials of guadecitabine for their DNA methylation status at baseline and following treatment, for baseline genetic mutations in a panel of 54 genes commonly mutated in hematopoietic malignancies, and for expression of a selected gene panel. Our data suggest that specific gene expression, DNA methylation, and mutational signatures may be associated with OS and therapy resistance in guadecitabine-treated patients.

## Materials and methods

### Patients

The phase I/II trials of guadecitabine were conducted under one study protocol and is registered with ClinicalTrials.gov (NCT01261312). The patients’ eligibility criteria in the phase I and the phase II stage were previously published [8, 10]. The patients evaluated in this report are those with r/r AML who were treated in the phase I stage on the 5× daily (≥ 30 mg/m^2^) or 3× weekly schedule (≥ 60 mg/m^2^) at therapeutic doses [8] and all those treated in phase II (60 and 90 mg/m^2^ both in a 5-day schedule and 60 mg/m^2^ in a 10-day schedule) [10] provided they had pre-treatment blood and/or bone marrow available for molecular analyses (Additional file 1 Table S1). Responses were evaluated by the IWG criteria [11]. Patients were considered responders if they had a composite complete response (CRc) including complete response (CR), CRp (CR with incomplete platelets recovery), or CRi (CR with incomplete blood count recovery). There were no statistically significant differences in the response rate or survival between the different doses/schedules [10].

### Baseline gene expression analysis

Whole-blood RNA was isolated and purified with the PAXgene Blood RNA Kit (Qiagen, Hilden, Germany) and QIAcube automated sample preparation system (Qiagen) by Covance Central Laboratory Services (Indianapolis, IN, USA), according to the manufacturer’s instruction. Any contaminated DNA from RNA preparations was removed with TURBO DNA-free kit (Ambion, Carlsbad, CA, USA) before cDNA synthesis. First-strand cDNA was synthesized using High-Capacity cDNA Reverse Transcription Kits (Applied Biosystems, Grand Island, NY, USA). The quantitative expression of a panel of genes (CDA, CDKN2B (P15), CDKN1A (P21), DNMT1, DNMT3A, DNMT3B, and CCCTC-binding factor (CTCF)) at baseline was performed by TaqMan® probe-based gene expression analysis by Applied Biosystems (Additional file 2 Table S2). These were selected because they are known epigenetic regulators (DNMT1, DNMT3A, DNMT3B, and CTCF), epigenetically regulated genes (P21 and P15), or determinant of decitabine levels (CDA). Human GAPDH was used as a normalization control in qPCR reactions. All qPCR reactions were run in triplicate and the values averaged. Negative controls where the reverse transcriptase enzyme was omitted were included in parallel to exclude the possibility of genomic DNA contamination. Relative target gene expression was represented by dCT (target gene) = Ct (GAPDH) − Ct (target gene). Unsupervised hierarchical clustering analysis of baseline gene expression in the patients was performed by ArrayTrack (the National Center for Toxicological Research) using the Ward method [12].

### DNA methylation profiling

Pre-treated whole-blood genomic DNA was purified with the QIAamp DNA Blood Mini Kit (Qiagen) and QIAcube automated sample preparation system (Qiagen) by Covance Central Laboratory Services. To analyze the genome-wide methylation profile, we used Digital Restriction Enzyme Analysis of Methylation (DREAM) [13, 14]. Briefly, DREAM methylation analysis is a quantitative mapping of DNA methylation with high resolution on a genome-wide scale without bisulfite conversion. The method is based on sequential cuts of genomic DNA with a pair of neoschizomer endonucleases recognizing the same restriction site (CCCGGG) containing a CpG dinucleotide. The first enzyme, *Sma*I, cuts only at the unmethylated CpG sites and leaves blunt ends. The second enzyme, *Xma*I, is not blocked by methylation and leaves a short 5′ overhang. The enzymes thus generate distinct methylation-specific signatures at the ends of restriction DNA fragments. These are deciphered by next-generation sequencing. Methylation level at individual CpG sites is calculated as the ratio of sequencing reads with the methylated signature to the total number of reads mapping to the site. Using the DREAM method, we analyzed pre-treatment DNA methylation profiles of the r/r AML patients (*n* = 116) from the phase I and II trials. Paired-end sequencing of 40 bases was performed on HiSeq 2500 (Illumina, San Diego, CA, USA) instrument at the Genomic Core Facility of Fox Chase Cancer Center (Philadelphia, PA, USA). These sequence data have been submitted to the GEO database under accession number GSE112838. We mapped the sequences to the human genome (hg19) and calculated the methylation at target sites as the fraction of reads with methylated signature. We included in the methylation analysis 17,793 CpG sites covered with at least 20 sequencing reads in at least 75% of samples. We imputed missing values (1.8% of total) by predictive mean matching [15] using the mice package in R suite [16]. Unsupervised hierarchical clustering (Ward.D) and heatmap were performed by the pheatmap R package. CpG site permutations were used to detect differentially methylated sites associated with response to guadecitabine. We used Ingenuity Pathway Analysis (Qiagen) for analyzing enriched canonical pathways of 121 genes with differentially methylated genes with CR and (separately) CRc in 116 patients. LINE-1 methylation dynamics were available on these patients from previous reports [8, 10].

### AML mutational profiles

Pre-treatment blood or bone marrow-derived DNA was available for analysis from a total of 122 patients with AML enrolled on guadecitabine phase I/II trials. We performed mutation analysis by targeted high-throughput sequencing using the TruSight Myeloid Sequencing Panel (Illumina). The panel covers 54 genes commonly mutated in hematologic malignancies. FLT3-ITD was difficult to detect using this panel; we also detected this mutation by PCR followed by capillary electrophoresis (LabPMM, San Diego, CA, USA). Sequencing library preparation was performed according to the manufacturer’s instruction. Paired-end sequencing of 2 × 150 bases was performed on the HiSeq 2500 instrument (Illumina), and reads were aligned to the human genome (hg19). Initial variants were called using the BaseSpace TrueSeq Amplicon app and BaseSpace Variant Studio 2.2 (Illumina). In addition, variants were required to have a minimum of 100 reads and a sequence quality score of at least 50. A minimum allelic fraction of 5% was set to identify somatic variants. Finally, we filtered the variants for presence in the COSMIC database (v78) with a hematopoietic malignancy association and absence from dbSNP (common variants) with a normal population frequency of > 1%.

### Statistical analysis

Fisher’s exact test was used to compare the differences in the CRc or CR rates. Unpaired *t* test or one-way ANOVA was used to compare the age, white blood cell (WBC) count, peripheral blood (PB) blast, and bone marrow (BM) blasts at screening in different (2 or 3) groups. We derived the maximum LINE-1 demethylation for patients during the first cycle of guadecitabine treatment. The mean maximum LINE-1 demethylation was compared between the groups using the Mann-Whitney test. Overall survival (OS) was measured as the time from the date of the first treatment to death (failure) or alive at last follow-up (censored). OS curves were estimated using the Kaplan-Meier method and compared between the groups using a log-rank test. To build a multivariate model of response to guadecitabine or OS by baseline expression, we derived a *z*-score of gene expression based on 4 genes (z4), obtained by zCDA + zP15 + zCTCF − zDNMT3B formula (based on gene expression clustering). Binary logistic regression and the Cox regression model were used for the univariate and the multivariate analysis, respectively, for 116 patients with mutational status and gene expression data to determine clinically meaningful differences in the achievement of CR, CRc, and OS. All *p* values are two-sided, and < 0.05 was considered significant. All the above statistical procedures were performed with GraphPad Prism (version 5.04) and SAS/STAT software (version 9.4).

## Results

### Patients studied

Additional file 3 Table S3 describes the demographic and clinical characteristics of the 29 patients on the phase I trial (17 of 25 (68%)) patients treated at therapeutic levels on the 5× daily schedule and 12 of 18 (67%) patients on the 3× weekly schedule) and the 99 patients (of 103 patients treated or 96%) on the phase II trials analyzed molecularly. The two groups were broadly similar with a CRc rate of 17% in phase I and 23% in phase II. Out of those 128 patients, we successfully obtained genome-wide methylation data in 116 (91%), mutation data in 122 (95%), and gene expression data in 122 (95%) (Additional file 1: Table S1).

### Genome-wide DNA methylation analysis

**Fig. 1:**
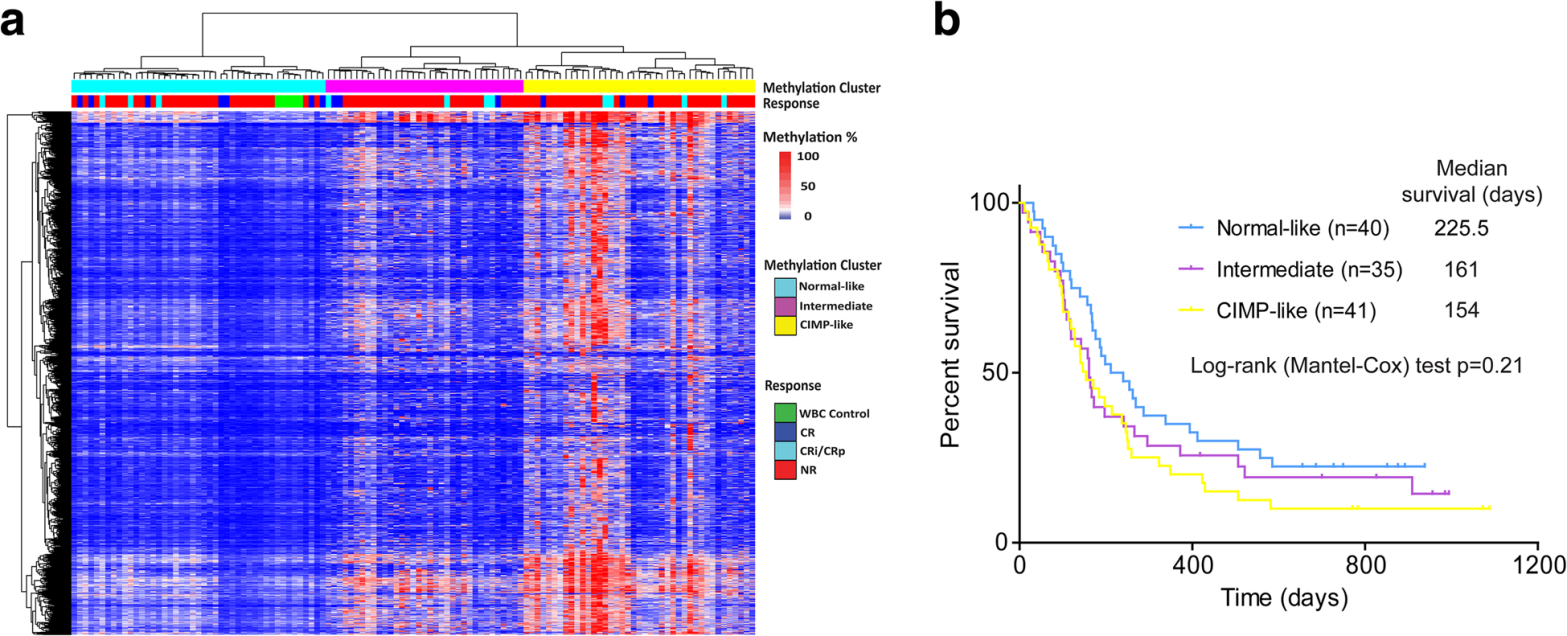
DNA methylation and response to guadecitabine. **a** Unsupervised hierarchical clustering of 116 r/r AML patients based on 2774 hypervariable sites (standard deviation > 10%) divided the cases into three clusters which were normal-like, CIMP-like, and intermediate methylation. **b** Kaplan-Meier survival analysis of 116 cases based on the clusters derived in **a**. There was a trend for longer survival in the “normal-like” cluster, but this trend was not statistically significant (*p* = 0.21 by log-rank test)

DREAM analysis of 17,793 sites in 116 patients identified 2774 hypervariable methylation sites (standard deviation > 10%). Unsupervised hierarchical clustering of these sites divided the cases into three clusters (Fig. 1a). Cluster 1 contained all five samples of normal blood mononuclear cells analyzed and was therefore called “normal-like.” Cluster 3 had more intense CpG island methylation reminiscent of the CpG island methylator phenotype (CIMP) [5, 17] and was therefore “CIMP-like,” while cluster 2 was characterized by intermediate methylation. Clinical characteristics of the three clusters are shown in (Additional file 4: Table S4). There were trends for higher CR rate and longer survival (Additional file 4: Table S4 and Fig. 1b) in the “normal-like” cluster, but these trends were not statistically significant.

We next analyzed the correlations between methylation at individual CpG sites and CR or CRc rates in a two-step process: discovery in the 27 patients on the phase I trial and validation in the 87 patients on the phase II trial. There were only 29 sites out of 17,793 analyzed that were associated with CR in both phase I and II patients at a *p* < 0.05 in both cohorts. We next combined data on all 116 patients and analyzed the correlations between methylation and CR in all. In this combined analysis, 879 CpG sites showed statistically significant (empirical *p* < 0.05 based on permutation testing) correlations with CR rate (and 459 sites with CRc rate). Among these, there was a significant enrichment for the sites in CTCF binding, predicted enhancer, and CpG islands (Additional file 5: Table S5). The sites included 121 genes with differentially methylated promoters, and Ingenuity Pathway Analysis of these is shown in (Additional file 6: Table S6). The most enriched pathway was the eicosanoid signaling pathway which is related to infection and inflammation. However, these data need validation in different sets of patients as a more stringent statistical test (FDR < 0.01) revealed no differences between responders and non-responders.

### Correlations between mutation status and response

**Fig. 2:**
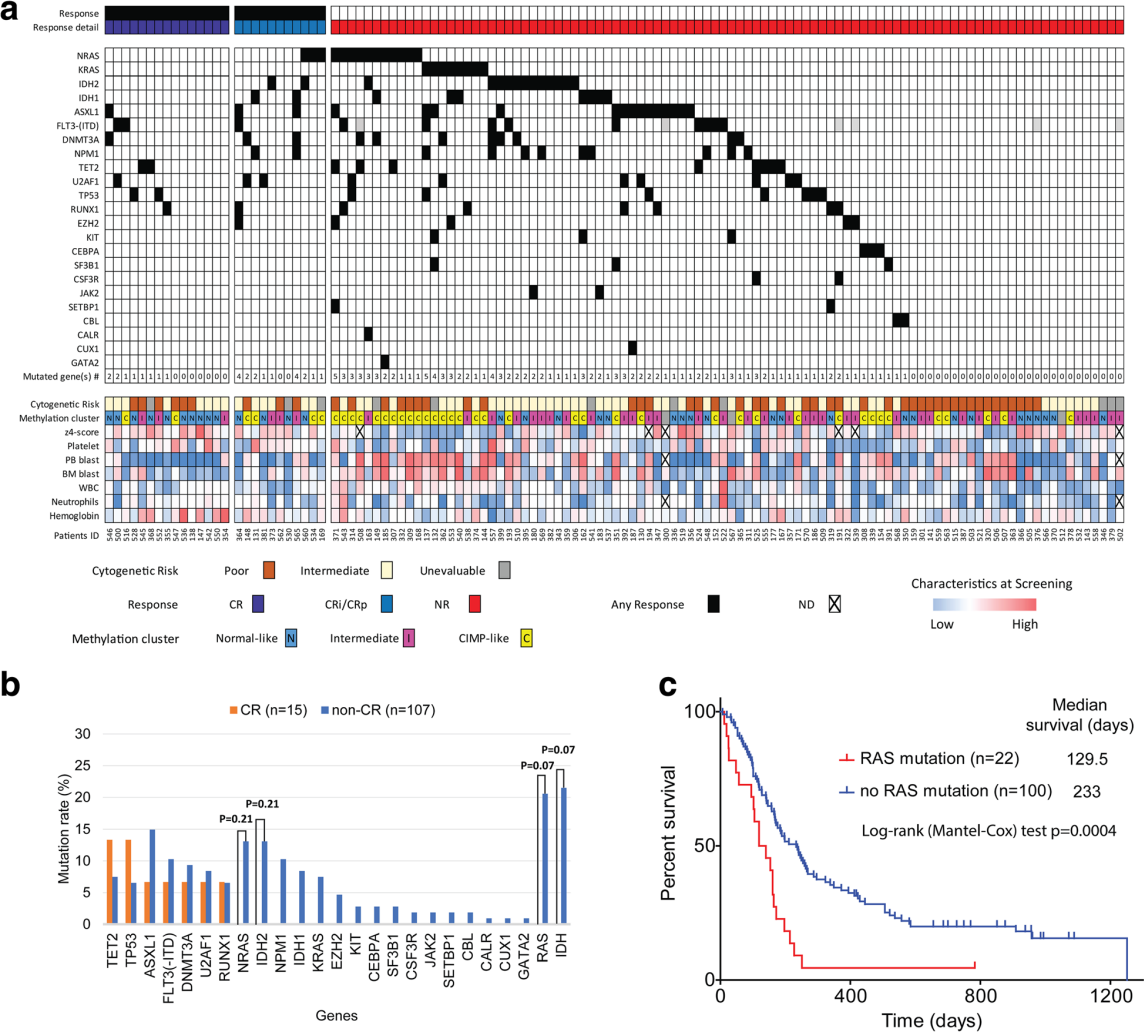
Mutation spectrum and response to guadecitabine. **a** Association of mutation spectrum, clinical characteristics, and response to guadecitabine in r/r AML (*n* = 122). Genomic mutation analysis was performed using the TruSight Myeloid Sequencing Panel (Illumina). The gene rows in the graph represent individual genomic lesions, the clinical characteristics row represent simplified clinical information, and the columns represent patients in the study. Black in the gene row indicates the presence of a specified mutation in a patient, and colors in the clinical characteristics row represent low (blue) to high (red). **b** Comparison of the mutation rate of CR vs. non-CR patients. None of the genes showed a significant correlation with CR, but there was a strong trend for RAS mutations (N or K) to be associated with resistance to guadecitabine (CR were seen in 0/22 patients with RAS mutations compared to 15/100 patients without RAS mutations, *p* = 0.07). **c** Kaplan-Meier survival analysis stratified by RAS mutation status. The presence of RAS mutations was associated with a significantly worse survival (*p* = 0.0004 by log-rank test)

We next queried for mutation status in a panel of 54 genes using the TruSight myeloid sequencing panel which analyzes a total of 338 exons (or 568 amplicons) by targeted deep sequencing. We successfully obtained data on a median aligned read of 94% (range 48–98%) in 122 patients (Fig. 2a). The median number of reads/amplicons was 9125 (range 3646 to 25,050). Overall, the median mutation number was 1 per case (range 0 to 5). We compared the mutation frequencies for the 54 genes in the 122 r/r AML patients with those reported for treatment-naïve AML (tn-AML) in the TCGA [3] and found significant differences at ASXL1, FLT3 (-ITD), DNMT3A, and NPM1 gene by the Bonferroni correction (*p* < 0.0026 to be significant after the Bonferroni adjustment of a major 19 genes comparison) (Additional file 7: Table S7). None of the genes showed significant correlations between mutations and CRc (Additional file 8: Figure S1). When we analyzed the correlations with CR, none of the genes showed a significant correlation either (Fig. 2b). No gene showed synthetic lethality as evidenced by a high response rate among cases with mutations, though there were minor non-significant trends for TET2 (CR rate of 2/10 (20%) in cases with mutations vs. 13/112 (12%) in cases without, *p* = 0.35) and TP53 (CR rate of 2/9 (22%) in cases with mutations vs. 13/113 (12%) in cases without, *p* = 0.31). Multiple genes were mutated at a higher frequency in those patients who did not respond (Fig. 2b), though none reached statistical significance. These included KRAS, NRAS, IDH1, IDH2, and multiple others. As previously reported, KRAS and NRAS mutations were mutually exclusive, and there was a strong trend for RAS mutations (N or K) to be associated with resistance to guadecitabine (CR cases were seen in 0/22 (0%) patients with RAS mutations compared to 15/100 (15%) patients without RAS mutations, *p* = 0.07). The presence of RAS mutations was associated with a more aggressive clinical phenotype and more methylated (CIMP-like and intermediate) clusters (Table 1) and a significantly worse survival (Fig. 2c, median survival 129.5 days with RAS mutation vs. 233 days without RAS mutation, log-rank test *p* = 0.0004). IDH1/2 mutations also were associated with a trend for resistance to guadecitabine (CRs were seen in 0/23 (0%) patients with IDH1/2 mutations compared to 15/99 (15%) patients without IDH1/2 mutations, *p* = 0.07). The presence of IDH2 mutation was associated with intermediate-risk cytogenetics (all 14 cases) and a significantly longer survival (Additional file 9: Figure S2, median survival 521 days with IDH2 mutation vs. 172 days without IDH2 mutation, log-rank test *p* = 0.03). In the case of patients with IDH1 mutation, there was no difference in survival (median survival 240 days with IDH1 mutation vs. 176 days without IDH1 mutation, log-rank test *p* = 0.53).

**Table 1:** Mutation status in RAS and methylation status and clinical characteristics

Characteristics	Mutation in RAS (*n* = 22)	No mutation in RAS (*n* = 100)	*p* value
Median age (range)	54 (29.1–81.7)	62 (23.4–86.1)	0.14
Sex			0.48
Male (%)	15 (68%)	59 (59%)	
Female (%)	7 (32%)	41 (41%)	
Cytogenetic risk			1
Poor (%)	9 (41%)	42 (42%)	
Intermediate (%)	10 (45%)	50 (50%)	
Unevaluable (%)	3 (14%)	8 (8%)	
PB blasts at screening (%), median (range)	58 (0–99)	5.5 (0–93)	< 0.0001
BM blasts at screening (%), median (range)	49 (18–95)	31.1 (2–94)	0.0005
Platelet count (K/μL) at screening, median (range)	31.5 (7–230)	36 (2–342)	0.44
Hemoglobin (g/dL) at screening, median (range)	9.4 (7.2–12.6)	9.3 (6.0–14.4)	0.96
WBC (K/μL) at screening, median (range)	9.5 (1.5–36.2)	2.1 (0.2–75.5)	0.0007
LINE-1 maximum demethylation %, mean ± SE*	18.9 ± 3.2	24.3 ± 1.3	0.056
IDH mutation	5 (23%)	18 (18%)	0.56
Methylation cluster			0.0009
CIMP-like	10 (45%)	31 (31%)	
Intermediate	11 (50%)	24 (24%)	
Normal-like	0	40 (40%)	
Unknown	1 (5%)	5 (5%)	
Response			
Complete CR rate (%)	0	15/100 (15%)	0.07
CRc rate (%)	3/22 (14%)	23/100 (23%)	0.4
Median survival days (range)	129.5 (12–783+)	233 (7–1088+)	0.0004
2-year survival rate	1/22 (5%)	19/100 (19%)	0.12

*Maximum LINE-1 demethylation for patients during the first cycle of guadecitabine treatment

### A gene expression predictor of resistance to guadecitabine

**Fig. 3:**
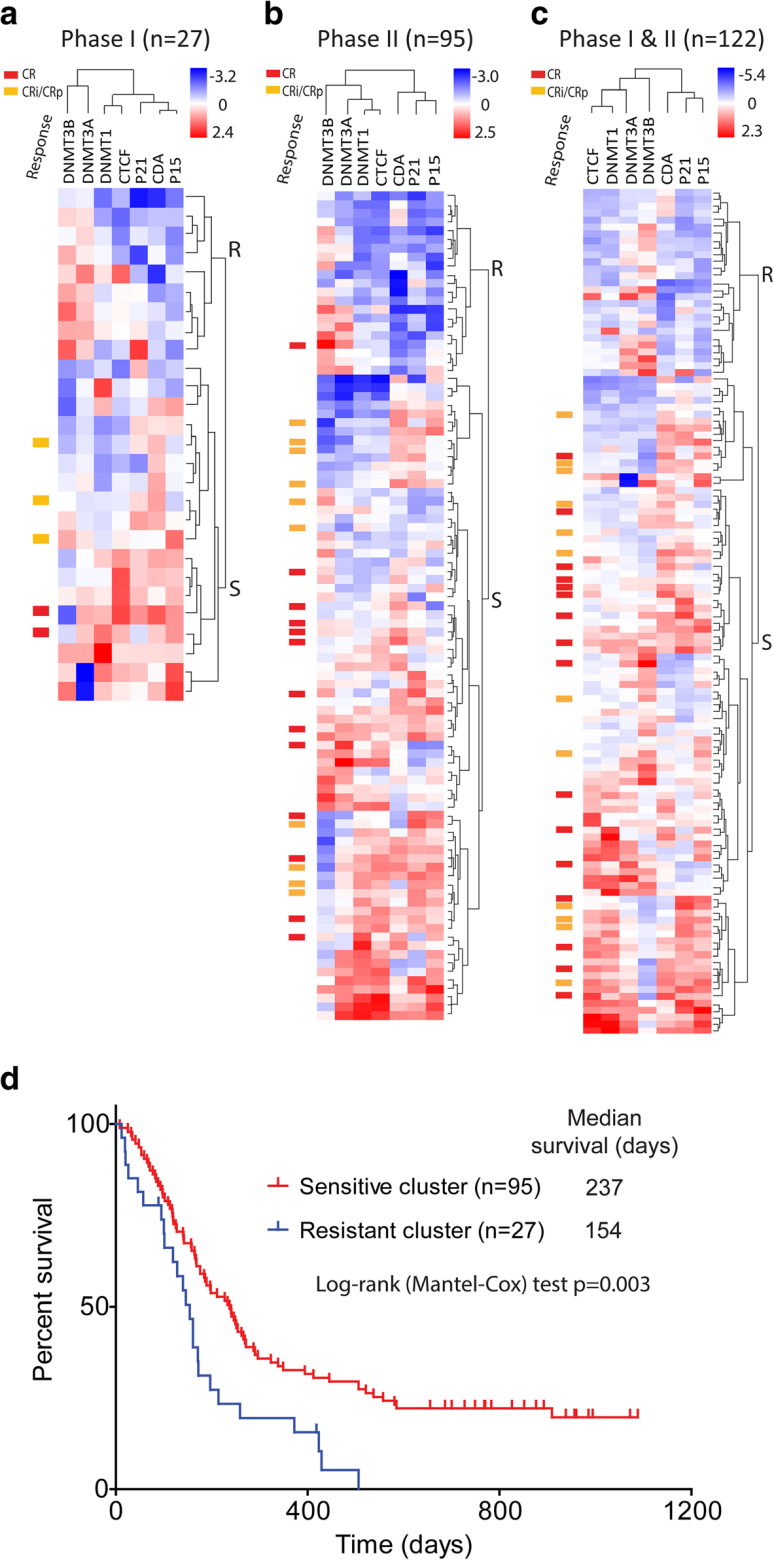
Selected gene expression profile and response to guadecitabine. **a** Unsupervised hierarchical clustering by baseline expression of the 7 gene panel grouped the phase I patients into two clusters (*n* = 27). Cluster R patients were clearly resistant to guadecitabine, with CRc seen in 0/9 patients compared to 5/18 patients for cluster S (*p* = 0.14). **b** Similar analyses of phase II patients (*n* = 95). These cluster R patients also showed resistance to guadecitabine (CRc seen in 1/21 patients compared to 22/74 patients for cluster S (*p* = 0.02), thus confirming the initial data in phase I patients. **c** A combined analysis of all 122 patients refined the clusters; 27 patients (17.2%) were in cluster R and had lower responses to guadecitabine (CRc seen in 0/27 patients compared to 28/95 patients for cluster S (*p* = 0.0005)). **d** Kaplan-Meier survival analysis of all patients by the clusters derived in **c**. Cluster R (*n* = 27) had a significantly worse survival after guadecitabine treatment (*p* = 0.003 by log-rank test)

We next examined the expression of a panel of 7 genes as potential predictors of response to guadecitabine. We used the patients on the phase I trial for discovery and the patients on the phase II trial for validation. In the 27 patients on the phase I trial, there were clear trends for correlations between expression and response (not shown), but most striking was evidence of co-regulation of these genes and an unsupervised hierarchical cluster analysis uncovered two groups of patients (Fig. 3a). Cluster R (resistant) was mainly characterized by low expression of DNMT1 and P15 and high expression of both DNMT3A and DNMT3B. Most strikingly, cluster R patients were resistant to guadecitabine, with CRc seen in 0/9 (0%) patients compared to 5/18 (28%) patients for cluster S (sensitive) (*p* = 0.14). Clinically, patients in cluster R had evidence of more aggressive AML with higher WBC, BM blasts, and PB blast counts (Additional file 10: Table S8). Based on this discovery cohort, we analyzed the same panel of genes in the 95 patients on the phase II trial and used hierarchical cluster analysis (Additional file 11: Table S9). As seen in Fig. 3b, a very similar dichotomy was observed in the validation cohort with a cluster of patients (cluster R) having high levels of DNMT3A and DNMT3B and low levels of DNMT1 and P15. These patients also showed resistance to guadecitabine (CRc seen in 1/21 (5%) patients compared to 22/74 (30%) patients for cluster S, *p* = 0.02), thus confirming the initial data of the patients on the phase I trial. Patients in cluster R had worse survival after guadecitabine treatment (Additional file 11: Table S9, median survival 154 days in R cluster vs. 248 days in S cluster, log-rank test *p* = 0.0006, and 2-year survival rate 0/21 (0%) in R cluster vs. 18/74 (24%) in S cluster, Fisher’s exact test *p* = 0.01). Hierarchical cluster analysis of all 122 patients in a combined phase I and II cohorts (Fig. 3c) refined the clusters; 27 patients (17%) were in cluster R and had strikingly lower CR or CRc rates (CRc seen in 0/27 (0%) patients compared to 28/95 (29%) patients for cluster S, *p* = 0.0005). Patients in cluster R had higher WBC, BM blasts, and PB blast counts (Table 2). Patients in cluster R also had worse survival after guadecitabine treatment (median survival 154 days in R cluster vs. 237 days in S cluster, log-rank test *p* = 0.0028, and 2-year survival rate 0/27 (0%) in R cluster vs. 20/95 (21%) in S cluster, Fisher’s exact test *p* = 0.0065) (Fig. 3d and Table 2). We next used *z*-scores to derive quantitative surrogates for the gene expression clusters. A 4 gene *z*-score classifier (z4-score derived by zCDA + zP15 + zCTCF – zDNMT3B) discriminated well between clusters R and S and was also powerful at predicting CR or CRc. CRc rate was 2/38 (5%) at z4-score < 0 and 26/84 (31%) at z4-score ≥ 0 (Fisher’s exact test *p* = 0.0011, Additional file 12: Figure S3). To compare how much these results are specific to AML patients, we performed a validation analysis in tn-AML cohort [3]. The univariate Cox regression analysis showed high expression of DNMT3B was associated with worse OS (hazard ratio (HR) = 1.26, 95% CI 1.07–1.49, *p* = 0.005), but CTCF, P15 and CDA were not.

**Table 2:** Characteristics of patients by baseline gene expression cluster in combined phase I and phase II cohorts

Characteristics	Resistant cluster (*n* = 27)	Sensitive cluster (*n* = 95)	*p* value
Median age (range)	63 (29.1–83.4)	61 (23.4–86.1)	0.76
Sex			0.37
Male (%)	19 (70%)	56 (59%)	
Female (%)	8 (30%)	39 (41%)	
Cytogenetic risk			0.5
Poor (%)	14 (52%)	38 (40%)	
Intermediate (%)	12 (44%)	47 (49%)	
Unevaluable (%)	1 (4%)	10 (11%)	
PB blasts at screening (%), median (range)	72 (10–99)	4 (0–86)	< 0.0001
BM blasts at screening (%), median (range)	71 (16–95)	26 (2–90)	< 0.0001
Platelet count (K/μL) at screening, median (range)	25 (2–342)	41 (4–289)	0.7
Hemoglobin (g/dL) at screening, median (range)	9.3 (6.0–11.7)	9.2 (6.5–14.4)	0.33
WBC (K/μL) at screening, median (range)	9.9 (1–34)	1.8 (0–76)	0.0005
LINE-1 maximum demethylation %, mean ± SE	15.4 ± 2.3	26.3 ± 1.4	0.0002
RAS mutation	12/27 (44%)	9/89 (10%)	0.0002
IDH mutation	6/27 (22%)	17/89 (19%)	0.78
z4-score mean ± SD	− 2.4 ± 1.26	1.90 ± 1.85	< 0.0001
Methylation cluster			< 0.0001
CIMP-like	18 (67%)	23 (24%)	
Intermediate	9 (33%)	23 (24%)	
Normal-like	0	38 (40%)	
Unknown	0	11 (12%)	
Response			
CR rate (%)	0	16/95 (17%)	0.02
CRc rate (%)	0	28/95 (29%)	0.0005
Median survival days (range)	154 (12–506)	237 (8–1088+)	0.003
2-year survival rate	0	20/95 (21%)	0.0065

### An integrated analysis of resistance to guadecitabine

Of all the molecular studies described earlier, gene expression of a panel of 7 genes and the presence of RAS mutations were the most promising predictive markers, with both being associated with resistance to guadecitabine. As shown in Tables 1 and 2, these two molecular events were associated. Patients in the gene expression “resistance” cluster were characterized by a more aggressive clinical course (higher WBC, BM blasts, and PB blast counts) and had a higher incidence of RAS mutations. Interestingly, DNA methylation clusters showing CIMP-like and intermediate patterns were also enriched in the resistance cluster (*p* < 0.0001) while cytogenetics showed no differences. As expected, z4-scores of gene expression were dramatically different between clusters R and S (Table 2), and they were inversely correlated with WBC count (*r* = − 0.24), PB blast count (*r* = − 0.86), and BM blast count (*r* = − 0.62). Patients with RAS mutations had a lower z4-score (− 0.99 in case of RAS mutations vs. 1.39 in case of RAS wild type, *p* < 0.0001) but no significant differences in cytogenetics risk.

**Fig. 4:**
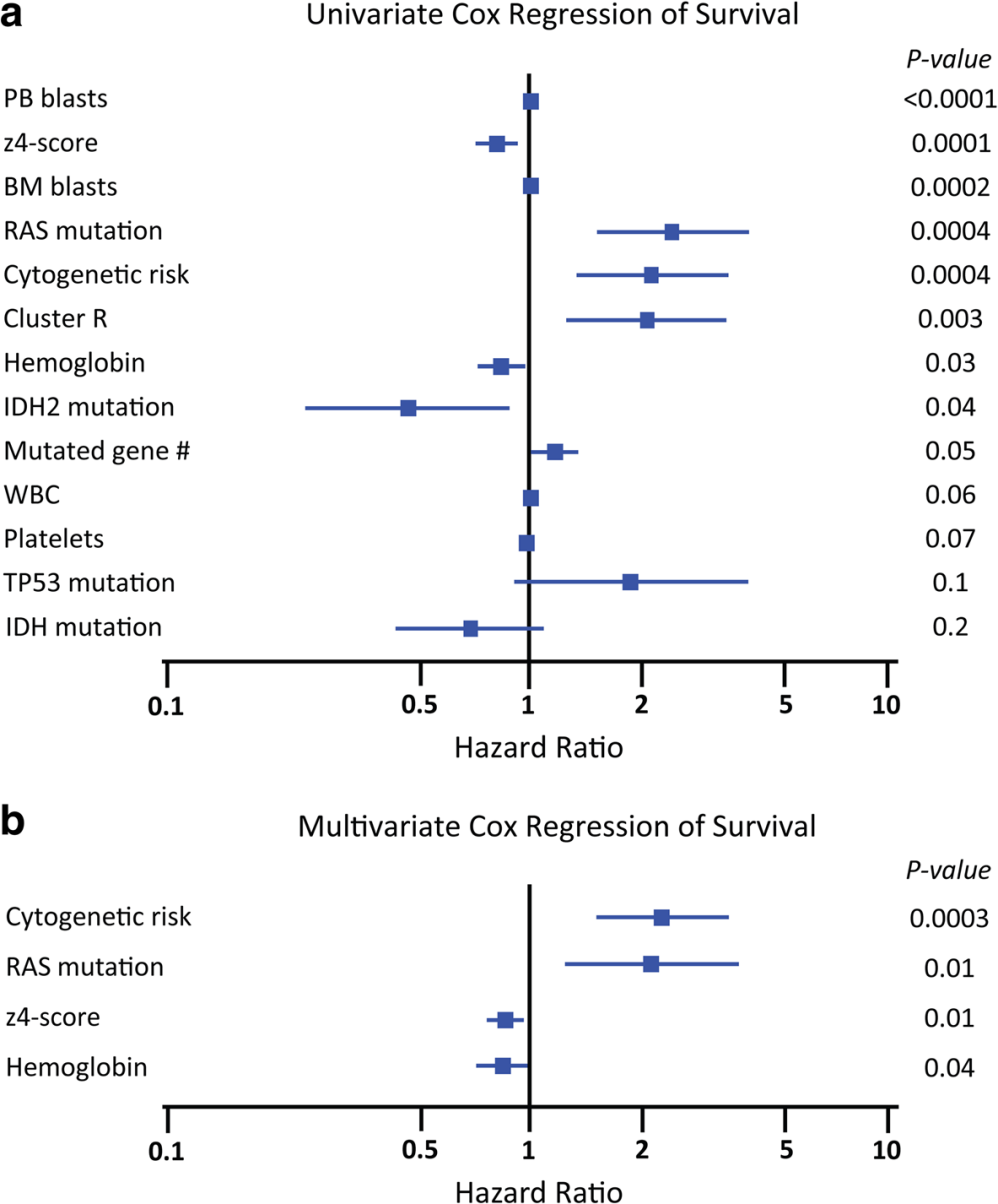
Univariate and multivariate COX regression to study survival after guadecitabine. **a** In univariate COX regression analyses, significant factors in univariate analyses were PB blasts (HR = 1.01, 95% CI 1.01–1.02, *p* < 0.0001), z4-score (HR = 0.85, 95% CI 0.78–0.91, *p* < 0.0001), BM blasts (HR = 1.02, 95% CI 1.01–1.02, *p* = 0.0002), RAS mutation (HR = 2.53, 95% CI 1.45–3.92, *p* = 0.0004), cytogenetic risk (HR = 2.18, 95% CI 1.41–3.17, *p* = 0.0004), cluster R (HR = 2.03, 95% CI 1.26–3.17, *p* = 0.003), hemoglobin value (HR = 0.86, 95% CI 0.75–0.99, *p* = 0.03), IDH2 mutation (HR = 0.47, 95% CI 0.23–0.98, *p* = 0.044), and mutated gene number (HR = 1.20, 95% CI 1.00–1.43, *p* = 0.048). **b** In a multivariate analysis by backward regression, cytogenetic risk (HR = 2.25, 95% CI 1.46–3.48, *p* = 0.0003), z4-score (HR = 0.89, 95% CI 0.81–0.97, *p* = 0.01), the presence of RAS mutation (HR = 2.12, 95% CI 1.19–3.76, *p* = 0.01), and hemoglobin value (HR = 0.86, 95% CI 0.74–0.99, *p* = 0.04) were significant predictors of survival

Towards an integrated model, we started with univariate logistic regression models for response (either CRc or CR). PB blasts, BM blasts, z4-score, and hemoglobin count were all significant, both CR and CRc at *p* < 0.01 (Additional file 13: Figure S4 and Additional file 14: Figure S5). In a multivariate model, hemoglobin level and PB blasts were significant predictors of CRc while hemoglobin level and z4-score were significant predictors of CR. The logistic regression model for CR could not be fitted for resistance clusters, IDH, or RAS mutation because there were no CR cases that were positive. We next examined survival. In a univariate Cox regression model, PB blasts, z4-score, BM blasts, RAS mutation, cytogenetic risk, and cluster of resistance were all significant at *p* < 0.01 (Fig. 4a and Additional file 15: Figure S6). In a multivariate Cox analysis, cytogenetic risk and the presence of RAS mutation were significant predictors of worse survival, while a higher z4-score and hemoglobin counts were significant predictors of longer survival (Fig. 4b).

Thus, a subset of patients with r/r AML characterized by a unique gene expression pattern, RAS mutations, high levels of CpG island methylation, high BM blasts, and high PB blasts is associated with poor response and poor survival after guadecitabine treatment. In multivariate analyses, a high hemoglobin level and low PB blasts were the best predictors of CRc, while gene expression (z4-score), cytogenetics, hemoglobin, and RAS mutations were all predictors of OS.

## Discussion

Guadecitabine (SGI-110) is a novel hypomethylating dinucleotide of decitabine and deoxyguanosine that is resistant to degradation by cytidine deaminase and results in prolonged in vivo exposure to its active moiety decitabine [8]. This differential pharmacokinetic profile offers the potential of improved biological and clinical activity and safety over currently available first-generation drugs such as azacitidine and decitabine. To develop genomic predictors of response to guadecitabine for r/r AML patients, we investigated hematopoietic cell samples from the patients enrolled in phase I/II trials of guadecitabine for their DNA methylation status, major genetic mutations, and a panel of gene expression at baseline. Our data suggested that there were no strong genetic, epigenetic, or gene expression predictors of CR or CRc to guadecitabine in r/r AML. From a DNA methylation perspective, the most enriched pathway of differentially methylated genes in responders to guadecitabine was the eicosanoid signaling pathway, possibly implying a role for inflammation and immunity [18] in these responses. However, this study suffers from potential overfitting effects and the data need to be validated in different population cohorts. The lack of correlation between global patterns of baseline DNA methylation and response is consistent with previous studies [19] and reflects the multifactorial nature of sensitivity to DNMT inhibitors [20].

From a genetic change perspective, the only genes with strong trends for (inverse) correlation with response were RAS (KRAS and NRAS) and IDH2. The data on IDH2 were surprising given that these patients have a strong hypermethylation phenotype [5, 21]; it is possible that the lack of TET function in these cases compromises the ability to stably demethylate and respond to HMAs. Interestingly, in a trial of an IDH2 inhibitor for r/r AML patients with IDH2 mutations, patients with NRAS mutations also had a worse prognosis [22]. The lack of an effect of TP53 mutations on response is different than what was observed with decitabine [23] and could reflect the fact that the population of patients treated here had relapsed/refractory disease. Of note, other studies have not found a correlation between TP53 mutations and response to HMAs [24]. Interestingly, the best molecular predictor of OS included gene expression of DNMT3B and CTCF which are well-known epigenetic regulators. CTCF is a zinc-finger DNA-binding protein that functions as transcriptional repressor or activator, as an insulator that can block the ability of enhancers to activate promoters, and as a three-dimensional (3D) chromatin organization. CTCF binding is determined by DNA sequence, methylation, and nucleosome occupancy [25]. DNMT3B and DNMT3A are responsible for establishing de novo DNA methylation patterns. DNMT3B and DNMT1 levels had opposite effects on the outcome after guadecitabine. From our validation analysis in the tn-AML cohort [3], we confirmed high expression of DNMT3B was associated with worse OS. Similar observations of worse clinical outcomes with high expression of DNMT3B were reported in de novo AML [26], pediatric AML [27], and older adult patients with cytogenetically normal AML [28]; this observation needs to be confirmed in genome-wide studies. In a mouse study, Dnmt3b, but not Dnmt3a, is upregulated following Dnmt1 deletion, and Dnmt3b is required for the survival of intestinal epithelium-specific Dnmt1-mutant mice [29]. In established HMA-resistant cell lines from a human monocytic leukemia cell line (MOLM-13), protein levels of DNMT3B were found upregulated compared to parental cell line and mRNA expression of DNMT1 and DNMT3A in HMA-resistant cell lines decreased compared to parental cell line [30]. In MDS-derived cell lines, the protein expressions of DNMT1 and DNMT3A, but not DNMT3B, tend to decrease in the presence of HMAs [31]. Additionally, cancer cell lines harboring DNMT3B gene amplification are less sensitive to the decrease in cell viability caused by HMAs [32]. Taken together, DNMT1, DNMT3A, and DNMT3B have different sensitivity to HMAs, and DNMT1 and DNMT3A, rather than DNMT3B, are the major targets of HMAs. And upregulation of DNMT3B could be a signature of resistance to HMAs. This baseline gene expression signature would be reflecting higher heterogeneity of HMA-resistant AML cells.

An integrated analysis identified a group of patients showing clear resistance to guadecitabine. They had a baseline expression signature of high-level expression of DNMT3B and low-level expression of P15, CTCF, and CDA; enrichment for KRAS or NRAS mutations; and a high degree of CpG island methylation. Clinically, these patients had higher bone marrow and peripheral blast counts, and interestingly, they had a significantly lower LINE-1 hypomethylation induction. Multivariate analysis also identified higher hemoglobin levels as a predictor of response and longer survival. It was reported that a hemoglobin value of at least 10 g/dL was associated with longer OS in patients with myelodysplastic syndromes treated with decitabine [33]. It is apparent therefore that the most aggressive relapsed/refractory AMLs are relatively refractory to single-agent guadecitabine, possibly because the pace of disease evolution overwhelms the ability of a slow-acting approach such as HMAs. If these patients could be identified prospectively, they may benefit from alternate approaches such as cytotoxic therapy, HMAs in combination with other therapies, or possibly a higher dose of HMAs. Indeed, it was reported that high-dose cytarabine treatment improves the responses in AML patients with RAS mutation [34]. Also, the phase III study of guadecitabine in r/r AML patients is using the 10-day regimen for up to 2 cycles with an allowance starting the second cycle earlier than 4 weeks in an attempt to provide a more myelosuppressive regimen upfront.

## Conclusions

We identified a subset of patients (~ 20% based on gene expression) with relapsed or refractory AML who are unlikely to show CR or CRc after a single-agent guadecitabine therapy. In the remaining 80% of patients without this poor prognosis signature, guadecitabine is an excellent option in r/r AML with a median survival of ~ 8 months and a 2-year survival rate of 21%. In the future, gene expression analysis and RAS mutation measurements may help in the development of effective personalized treatment strategies for patients with r/r AML.

## Additional files

Additional file 1: Table S1. Summary of studies reported in this paper (XLSX 8 kb)

Additional file 2: Table S2. List of TaqMan gene expression assays* used in this study. (XLSX 8 kb)

Additional file 3: Table S3. Demographic and baseline characteristics of the analyzed r/r AML patients in phase I and II (XLSX 11 kb)

Additional file 4: Table S4. Clinical characteristics of relapsed/refractory AML patients at baseline, survival, and response in the CGI methylation clusters (XLSX 11 kb)

Additional file 5: Table S5. Locations of 879 differentially methylated *Sma*I CpG sites correlated with complete response (CR) in the methylation analysis of a total of 17,793 CpG sites (≥ 20 reads) by DREAM analysis (empirical *p* < 0.05 based on permutation testing). (XLSX 10 kb)

Additional file 6: Table S6. Canonical pathway analysis (Ingenuity Pathway Analysis) of 121 genes with differentially methylated promoters from an analysis of 17,793 CpG sites (≥ 20 reads). (XLSX 8 kb)

Additional file 7: Table S7. Comparison of the major 19 mutated genes in this study and TCGA tn-AML (*n* = 200). (*p* < 0.0026 is significant after the Bonferroni adjustment) (XLSX 10 kb)

Additional file 8: Figure S1. Comparison of the mutation rate of composite complete response (CRc) to guadecitabine vs. non-response patients. None of the genes showed a significant correlation with CRc, but there was a trend for KRAS mutations to be associated with resistance to guadecitabine (CRc were seen in 0/26 patients with KRAS mutations compared to 8/96 patients without KRAS mutations, *p* = 0.2). (TIF 1237 kb)

Additional file 9: Figure S2. Kaplan-Meier survival analysis stratified by IDH2 mutation status. The presence of IDH2 mutations was associated with a significantly better survival (median survival 521 days with IDH2 mutation vs. 171.5 days without IDH2 mutation, log-rank test *p* = 0.03). (TIF 1167 kb)

Additional file 10: Table S8. Characteristics of r/r AML patients by baseline gene expression cluster in phase I (XLSX 11 kb)

Additional file 11: Table S9. Characteristics of r/r AML patients by baseline gene expression cluster in phase II (XLSX 11 kb)

Additional file 12: Figure S3. Waterfall plot of z4-score and response. We used *z*-scores to derive quantitative surrogates for the gene expression clusters. A 4 gene *z*-score classifier (z4-score derived by zCDA + zP15 + zCTCF − zDNMT3B) was powerful at predicting CR or CRc. CRc rate was 2/38 (5%) at z4-score < 0 and 26/84 (31%) at z4-score ≥ 0 (Fisher’s exact test *p* = 0.0011). (TIF 1032 kb)

Additional file 13: Figure S4. Univariate and multivariate logistic regression of composite complete response (CRc) to guadecitabine. In univariate logistic regression analyses of CRc, significant factors were hemoglobin value (odds ratio (OR) = 1.61, 95% CI 1.18–2.20, *p* = 0.003), z4-score (OR = 1.41, 95% CI 1.13–1.76, *p* = 0.003), PB blasts (OR = 0.96, 95% CI 0.94–0.99, *p* = 0.005), and BM blasts (OR = 0.97, 95% CI 0.95–0.99, *p* = 0.006). In a multivariate analysis by backward regression, hemoglobin value (OR = 1.56, 95% CI 1.14–2.15, *p* = 0.006) and PB blasts (OR = 0.96, 95% CI 0.93–0.99, *p* = 0.007) were significant predictors of response. (TIF 1009 kb)

Additional file 14: Figure S5. Univariate and multivariate logistic regression of complete response (CR) to guadecitabine. In univariate logistic regression analyses of CR, significant factors were hemoglobin value (OR = 1.84, 95% CI 1.27–2.5, *p* = 0.001), z4-score (OR = 1.48, 95% CI 1.11–1.98, *p* = 0.008), BM blasts (OR = 0.97, 95% CI 0.94–0.996, *p* = 0.03), and PB blasts (OR = 0.95, 95% CI 0.90–0.995, *p* = 0.03). In a multivariate analysis, hemoglobin value (OR = 1.70, 95% CI 1.18–2.46, *p* = 0.005) and z4-score (OR = 1.43, 95% CI 1.04–1.95, *p* = 0.03) were significant predictors of response. (TIF 948 kb)

Additional file 15: Figure S6. Univariate COX regression of z4 component genes. In univariate COX regression analyses of z4 component genes, the significant factors in univariate analyses were zCTCF (HR = 0.66, 95% CI 0.52–0.84, *p* = 0.0006), zDNMT3B (HR = 1.52, 95% CI 1.17–1.98, *p* = 0.002), zCDA (HR = 0.77, 95% CI 0.62–0.96, *p* = 0.02), and zP15 (HR = 0.82, 95% CI 0.66–1.02, *p* = 0.08). (TIF 1079 kb)

## Data Availability

The datasets generated and analyzed during the current study are available from the corresponding author on reasonable request. In addition, the DNA methylation profiles discussed in this publication have been deposited in NCBI’s Gene Expression Omnibus database under accession number GSE112838.

## Abbreviation

BM: Bone marrow
CDA: Cytidine deaminase
CIMP: CpG island methylator phenotype
CR: Complete remission
CRc: Composite complete response
CRi: CR with incomplete blood count recovery
CRp: CR with incomplete platelets recovery
CTCF: CCCTC-binding factor
DNMT: DNA methyltransferase
DREAM: Digital Restriction Enzyme Analysis of Methylation
HMA: Hypomethylating agent
IDH: Isocitrate dehydrogenase
LINE-1: Long interspersed nuclear element 1
MDS: Myelodysplastic syndromes
OS: Overall survival
P15: CDKN2B Cyclin-dependent kinase inhibitor 2B
P21: CDKN1A Cyclin-dependent kinase inhibitor 1
PB: Peripheral blood
r/r AML: Relapsed/refractory acute myeloid leukemia
TET: Tet methylcytosine dioxygenase
tn-AML: Treatment-naïve acute myeloid leukemia
WBC: White blood cell
