# Supplementary figures and images for: Genomic and epigenomic predictors of response to guadecitabine in relapsed/refractory acute myelogenous leukemia
Source: Clin Epigenetics. 2019 Jul 22;11:106. doi: 10.1186/s13148-019-0704-3 (PMC6647096; doi:10.1186/s13148-019-0704-3)

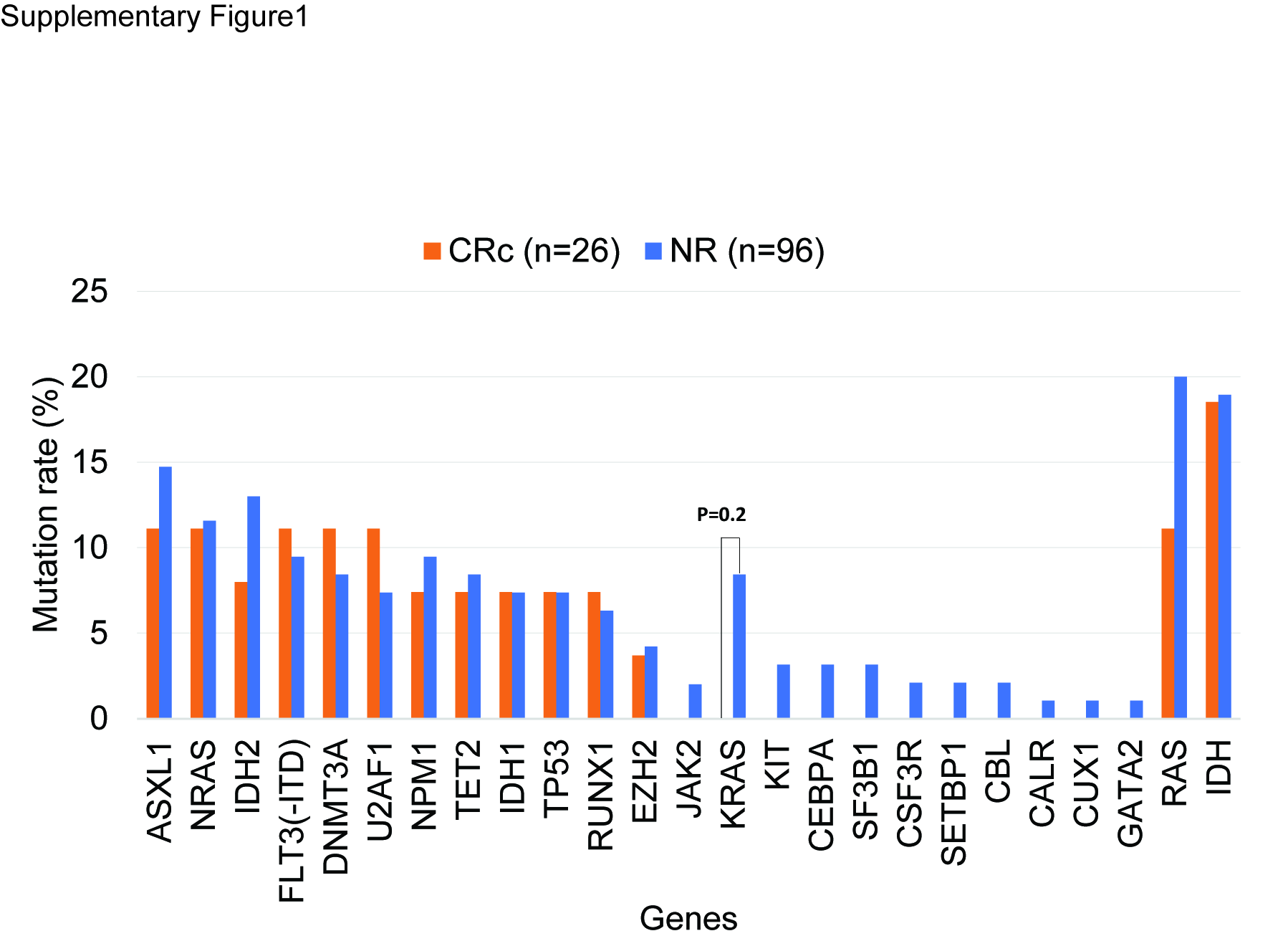

Supplement: Supplementary file 8 — Figure S1. Comparison of the mutation rate of composite complete response (CRc) to guadecitabine vs. non-response patients. None of the genes showed a significant correlation with CRc, but there was a trend for KRAS mutations to be associated with resistance to guadecitabine (CRc were seen in 0/26 patients with KRAS mutations compared to 8/96 patients without KRAS mutations, p = 0.2). (TIF 1237 kb) [file 13148_2019_704_MOESM8_ESM.tif]

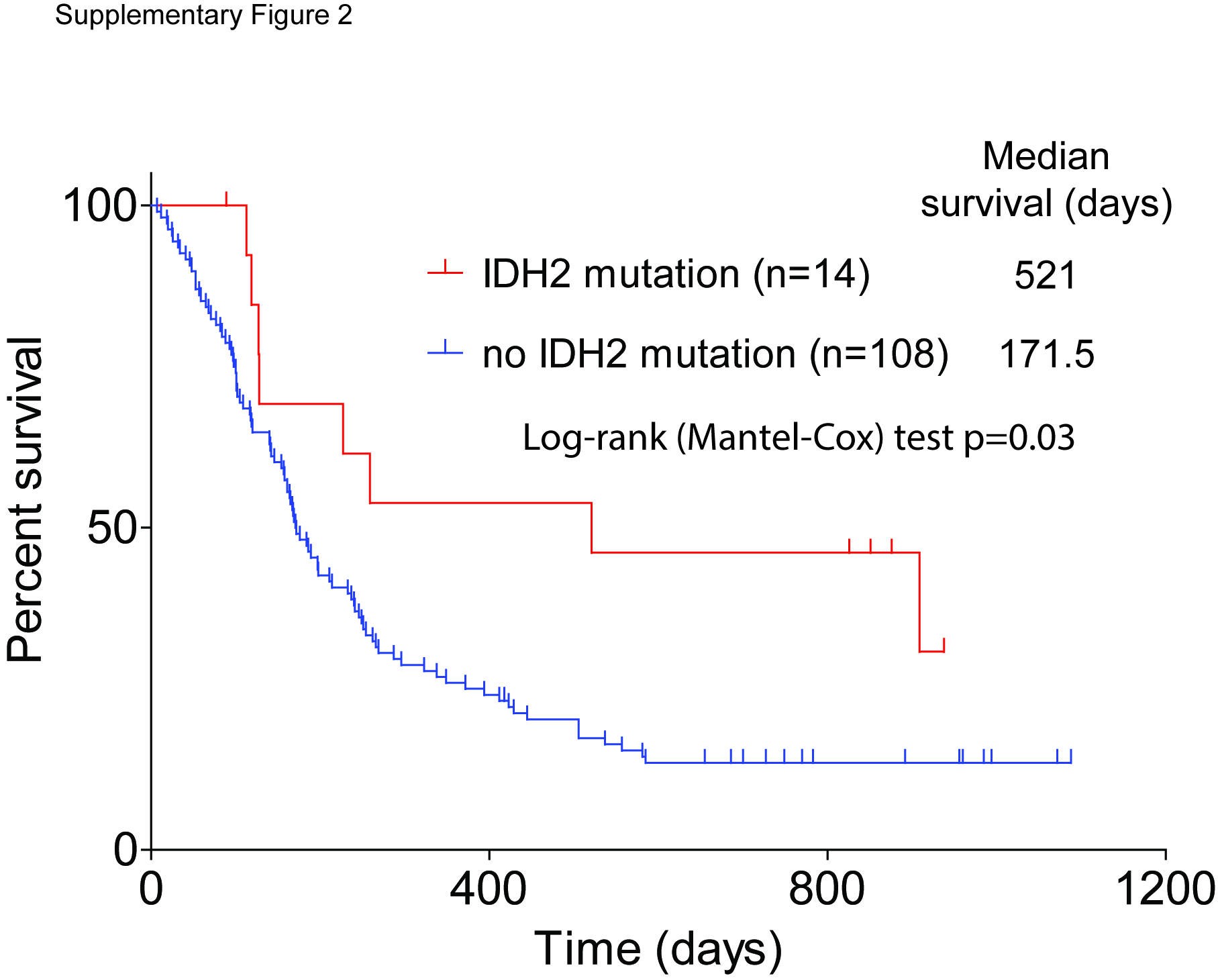

Supplement: Supplementary file 9 — Figure S2. Kaplan-Meier survival analysis stratified by IDH2 mutation status. The presence of IDH2 mutations was associated with a significantly better survival (median survival 521 days with IDH2 mutation vs. 171.5 days without IDH2 mutation, log-rank test p = 0.03). (TIF 1167 kb) [file 13148_2019_704_MOESM9_ESM.tif]

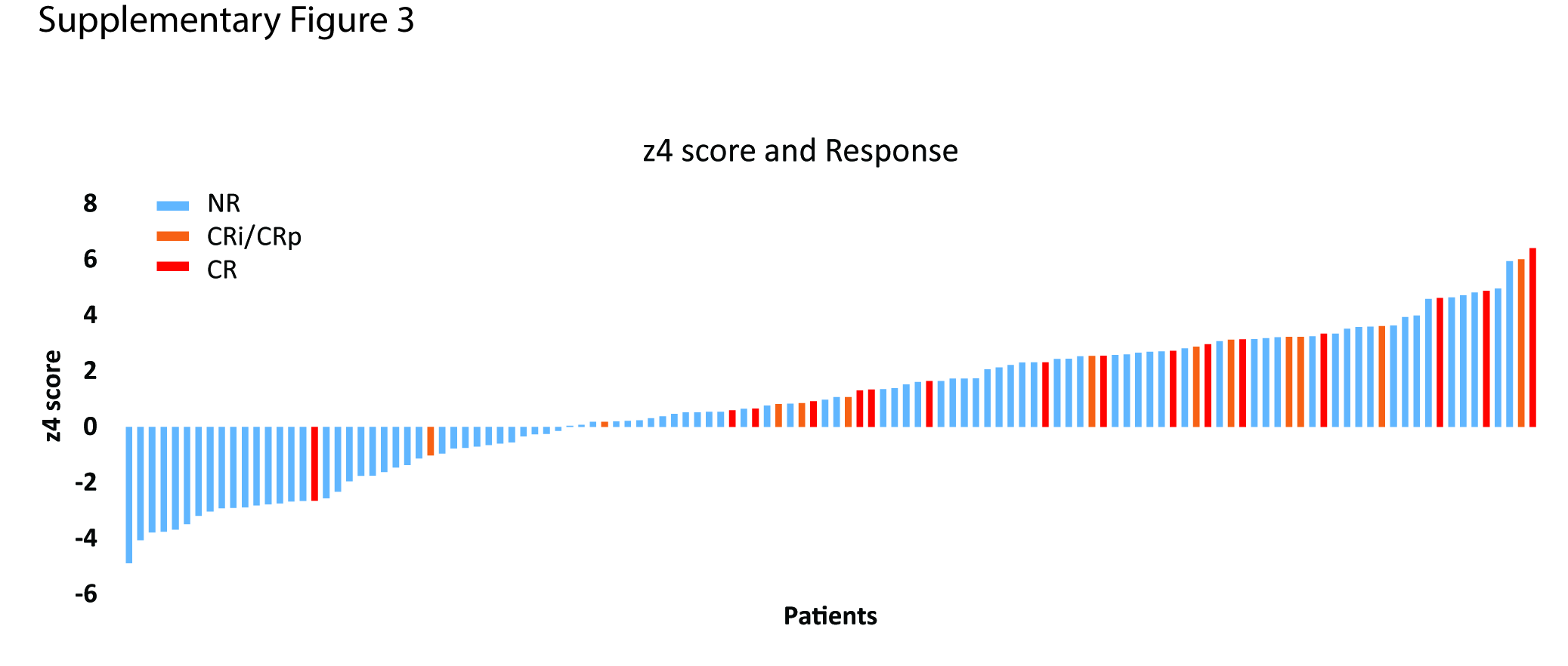

Supplement: Supplementary file 12 — Figure S3. Waterfall plot of z4-score and response. We used z-scores to derive quantitative surrogates for the gene expression clusters. A 4 gene z-score classifier (z4-score derived by zCDA + zP15 + zCTCF − zDNMT3B) was powerful at predicting CR or CRc. CRc rate was 2/38 (5%) at z4-score < 0 and 26/84 (31%) at z4-score ≥ 0 (Fisher’s exact test p = 0.0011). (TIF 1032 kb) [file 13148_2019_704_MOESM12_ESM.tif]

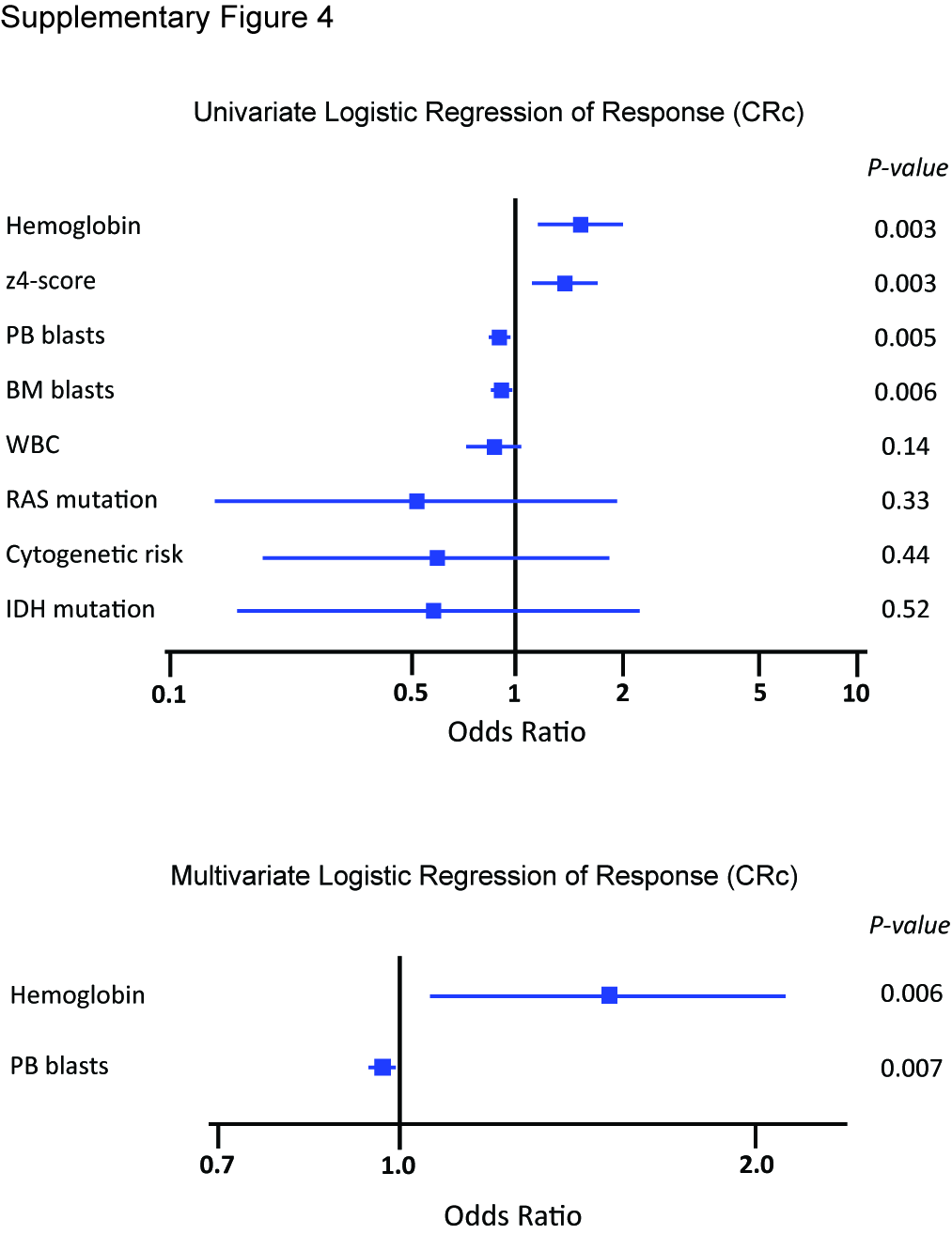

Supplement: Supplementary file 13 — Figure S4. Univariate and multivariate logistic regression of composite complete response (CRc) to guadecitabine. In univariate logistic regression analyses of CRc, significant factors were hemoglobin value (odds ratio (OR) = 1.61, 95% CI 1.18–2.20, p = 0.003), z4-score (OR = 1.41, 95% CI 1.13–1.76, p = 0.003), PB blasts (OR = 0.96, 95% CI 0.94–0.99, p = 0.005), and BM blasts (OR = 0.97, 95% CI 0.95–0.99, p = 0.006). In a multivariate analysis by backward regression, hemoglobin value (OR = 1.56, 95% CI 1.14–2.15, p = 0.006) and PB blasts (OR = 0.96, 95% CI 0.93–0.99, p = 0.007) were significant predictors of response. (TIF 1009 kb) [file 13148_2019_704_MOESM13_ESM.tif]

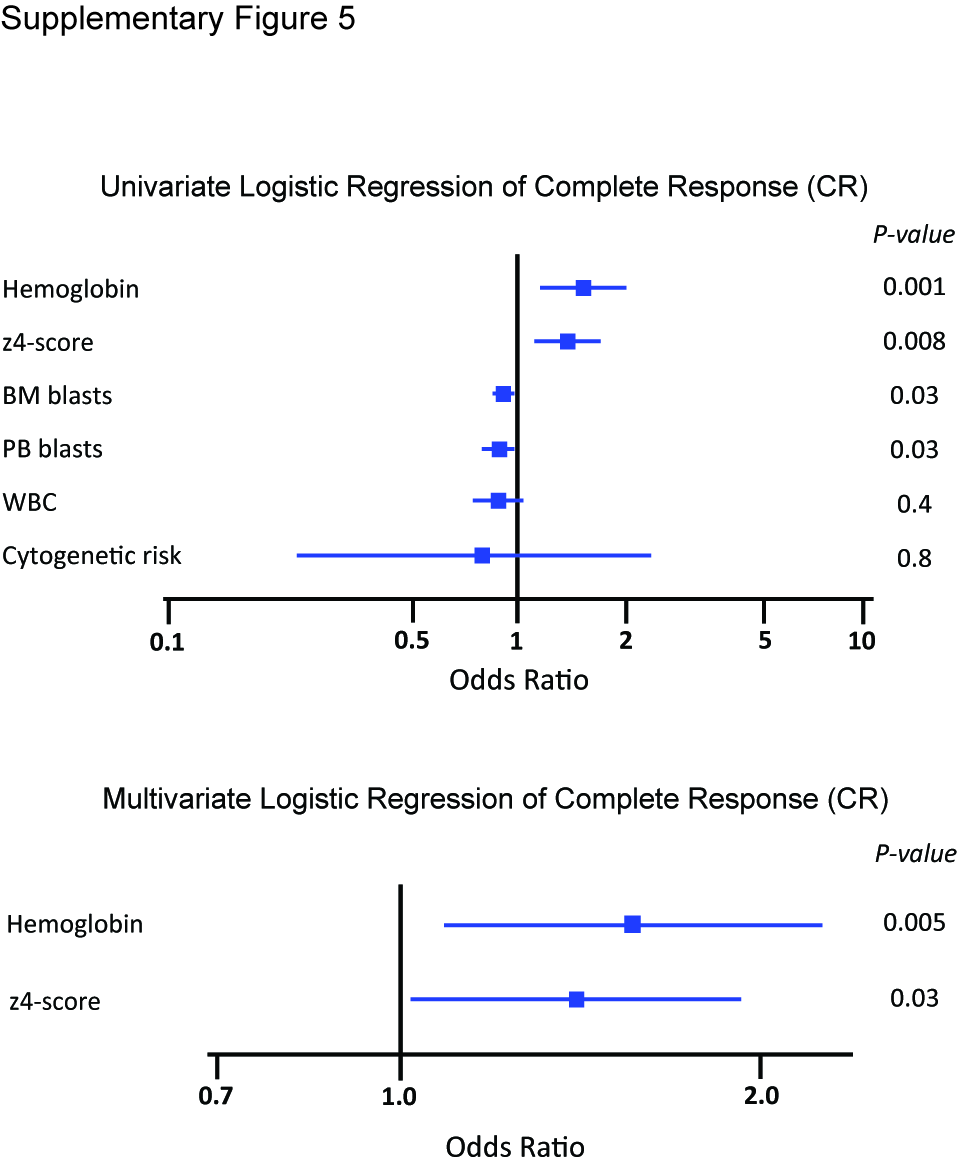

Supplement: Supplementary file 14 — Figure S5. Univariate and multivariate logistic regression of complete response (CR) to guadecitabine. In univariate logistic regression analyses of CR, significant factors were hemoglobin value (OR = 1.84, 95% CI 1.27–2.5, p = 0.001), z4-score (OR = 1.48, 95% CI 1.11–1.98, p = 0.008), BM blasts (OR = 0.97, 95% CI 0.94–0.996, p = 0.03), and PB blasts (OR = 0.95, 95% CI 0.90–0.995, p = 0.03). In a multivariate analysis, hemoglobin value (OR = 1.70, 95% CI 1.18–2.46, p = 0.005) and z4-score (OR = 1.43, 95% CI 1.04–1.95, p = 0.03) were significant predictors of response. (TIF 948 kb) [file 13148_2019_704_MOESM14_ESM.tif]

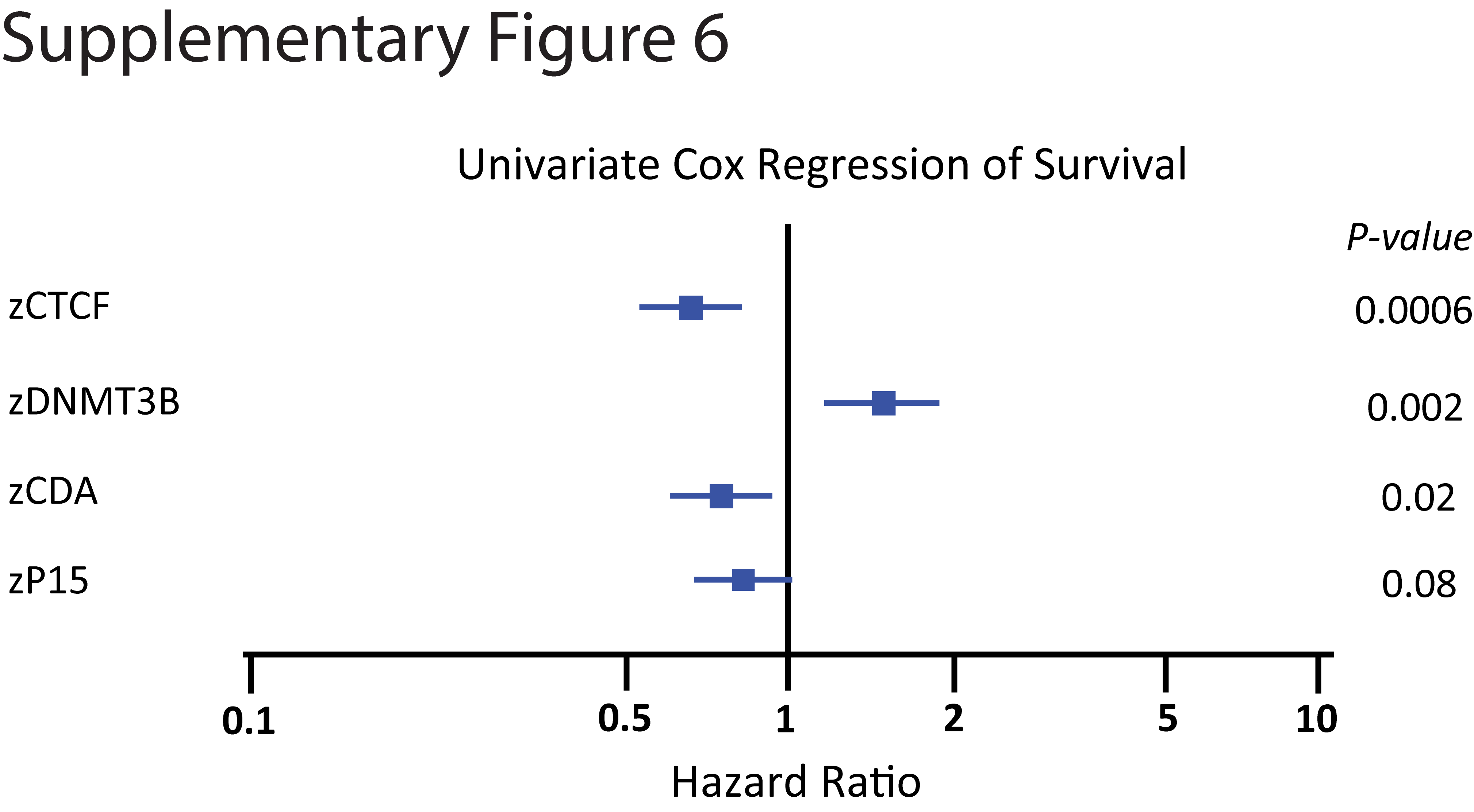

Supplement: Supplementary file 15 — Figure S6. Univariate COX regression of z4 component genes. In univariate COX regression analyses of z4 component genes, the significant factors in univariate analyses were zCTCF (HR = 0.66, 95% CI 0.52–0.84, p = 0.0006), zDNMT3B (HR = 1.52, 95% CI 1.17–1.98, p = 0.002), zCDA (HR = 0.77, 95% CI 0.62–0.96, p = 0.02), and zP15 (HR = 0.82, 95% CI 0.66–1.02, p = 0.08). (TIF 1079 kb) [file 13148_2019_704_MOESM15_ESM.tif]
